# Modified Epidermal Growth Factor Receptor (EGFR)-Bearing Liposomes (MRBLs) Are Sensitive to EGF in Solution

**DOI:** 10.1371/journal.pone.0007391

**Published:** 2009-10-09

**Authors:** Albert Wong

**Affiliations:** Program in Biological and Biomedical Sciences, Yale University, New Haven, Connecticut, United States of America; Johns Hopkins School of Medicine, United States of America

## Abstract

Cancers often overexpress EGF and other growth factors to promote cell replication and migration. Previous work has not produced targeted drug carriers sensitive to abnormal amounts of growth factors. This work demonstrates that liposomes bearing EGF receptors covalently crosslinked to p-toluic acid or methyl-PEO_4_-NHS ester (or, in short, MRBLs) exhibit an increased rate of release of encapsulated drug compounds when EGF is present in solution. Furthermore, the modified EGF receptors retain the abilities to form dimers in the presence of EGF and bind specifically to EGF. These results demonstrate that MRBLs are sensitive to EGF in solution and indicate that MRBL-reconstituted modified EGF receptors, in the presence of EGF in solution, form dimers which increase MRBL permeability to encapsulated compounds.

## Introduction

Cell replication is a fundamental process that maintains the human body in working order. Although this process is closely regulated under normal conditions, sometimes cells with deleterious genetic mutations may evade these checkpoints and replicate (rather than undergo apoptosis). If these cells and their daughter cells are not detected by the immune system, they may continue to replicate and accrue further deleterious mutations, a process that may, given time, lead to cancer [1]. Cancers often overexpress growth factors such as EGF to facilitate cell replication and migration [2].

Cancers that produce abnormal amounts of EGF, known as epidermal growth factor (EGF)-overexpressing cancers, have, along with other types of cancers, drawn enormous expense for treatment [1], [3], [4], [5]. Such treatment generally involves surgery, radiation therapy, or generalized chemotherapy, or some combination thereof. All of these treatment methods are attendant with significant systemic risks and side effects, and repeat treatments are often necessary [1], [6], [7]. Research toward developing better cancer therapies is of critical importance.

A newer approach for treating EGF-overexpressing cancers involves EGF receptor inhibitors (EGFRIs). Although EGFRIs represent an improvement over generalized chemotherapy in terms of efficacy and specificity, they often need to be used along with another treatment method (such as radiation therapy) and, further, are still attendant with potentially significant skin, hair, nail, and mucosal side effects [8], [9], [10].

The use of a targeted drug carrier (e.g., a liposome) to release chemotherapeutic drugs specifically in the neighborhood of EGF-overexpressing tumors has the potential to achieve the treatment ideality of maximal efficacy and maximal specificity [11]. Existing targeted drug carriers are generally triggered by factors such as ultrasound [12], [13]. To use these drug carriers *in vivo*, ultrasound waves (or other triggering factor) are aimed at tumors' precise locations so that drugs are only released from the carriers when they are at those locations [12], [13]. However, particularly for metastatic tumors, it is not always possible to identify the tumors' precise locations [1]. Without this information, it is not possible to achieve targeted drug delivery with these carriers. Hence, an ideal targeted drug carrier for EGF-overexpressing tumors would be one sensitive to abnormal amounts of EGF (i.e., an EGF-triggered carrier).

Here it is demonstrated *in vitro* that liposomes bearing EGF receptors crosslinked to p-toluic acid or methyl-PEO_4_-NHS ester (or, in short, MRBLs) are sensitive to EGF in solution.

## Results

### MRBLs are sensitive to EGF

**Figure 1:**
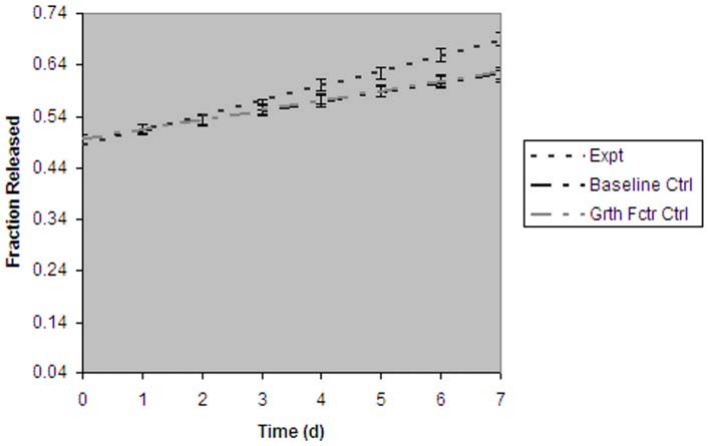
EGF in solution increases rate of encapsulated actinomycin D release. Percentage (expressed as fraction released) of encapsulated actinomycin D released as assessed by ^1^H NMR spectroscopy. Baseline Ctrl, actinomycin D-encapsulating MRBLs without growth factor added to solution. Grth Fctr Ctrl, actinomycin D-encapsulating MRBLs with VEGF added to solution. Expt, actinomycin D-encapsulating MRBLs with EGF added to solution.

Assessment of *in vitro* actinomycin D release from MRBLs demonstrated that the presence of EGF in solution resulted in an increased rate of actinomycin D release (Figure 1).

### EGF can induce dimerization of modified EGF receptors

**Figure 2:**
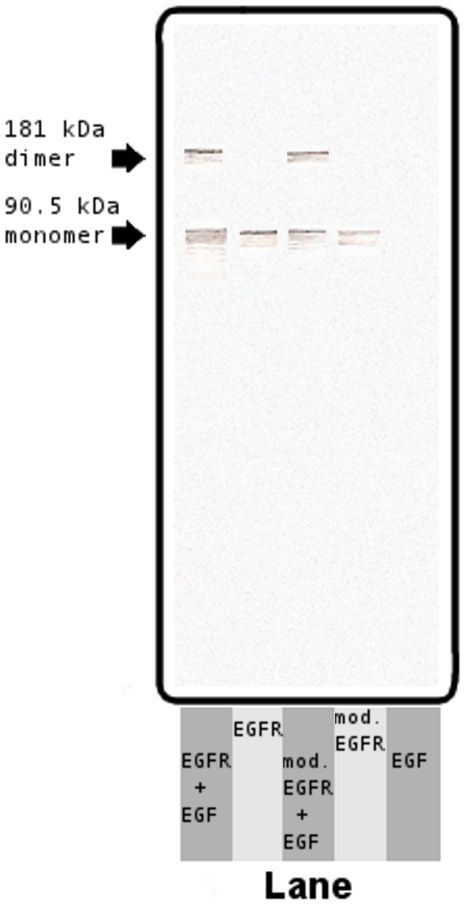
EGF induces dimerization of modified EGF receptors. SDS-PAGE analysis of EGFR dimerization. EGFR+EGF Lane, unmodified EGF receptors with EGF present. EGFR Lane, unmodified EGF receptors without EGF present. mod. EGFR + EGF Lane, modified EGF receptors with EGF present. mod. EGFR Lane, modified EGF receptors without EGF present. EGF Lane, only EGF present (no EGF receptors present).

It was next sought to verify whether EGF could induce dimerization of the modified EGF receptors. Modified EGF receptors and unmodified EGF receptors were assessed with and without the presence of EGF. In the presence of EGF, modified and unmodified EGF receptors formed dimers; in the absence of EGF, only receptor monomers were detected (Figure 2).

### MRBL-borne modified EGF receptors show specificity for EGF

**Figure 3:**
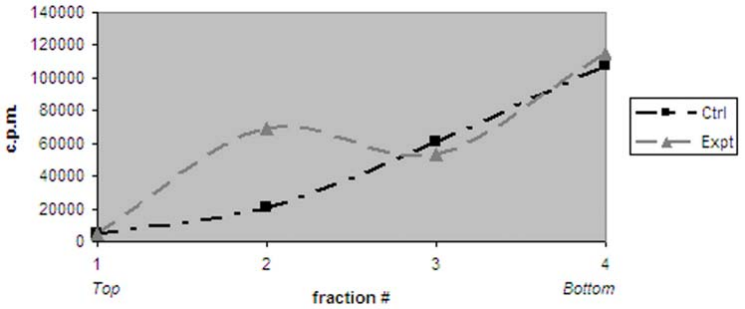
MRBL-borne modified EGF receptors show binding specificity for EGF. Binding of [^125^I]EGF to MRBLs as assessed by liquid scintillation counting. Ctrl, MRBLs pre-incubated with an excess of unlabeled EGF before incubation with [^125^I]EGF. Expt, MRBLs not pre-incubated with an excess of unlabeled EGF before incubation with [^125^I]EGF.

To assess whether MRBL-borne modified EGF receptors retain specificity for EGF, binding of radiolabeled [^125^I]EGF to reconstituted receptors was assessed with and without pre-incubation of the MRBLs with an excess of unlabeled EGF. [^125^I]EGF binding was observed only in the absence of unlabeled EGF pre-incubation (Figure 3).

### Modified EGF receptor dimers affect MRBL permeability to encapsulated drug

**Figure 4:**
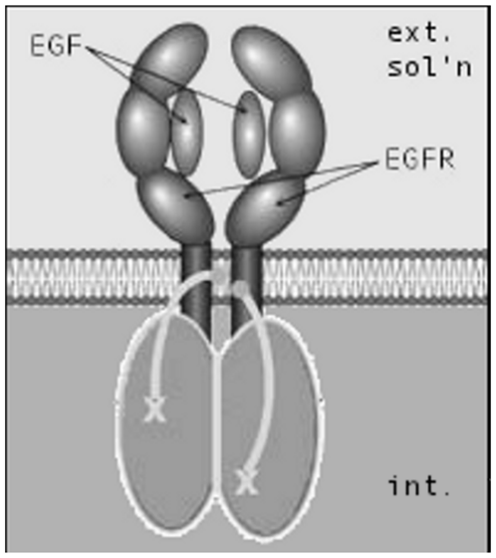
MRBL-borne modified EGF receptor dimers interact with the MRBL lipid bilayer to increase MRBL permeability (schematic). Dimerization of MRBL-borne modified EGF receptors. ext. sol'n, exterior solution. EGFR, modified EGF receptors. int., interior of unilamellar MRBL. X-----, crosslinked p-toluic acid or methyl-PEO_4_-NHS ester.

To identify the components underlying the EGF sensitivity of MRBLs, it was noted that modified EGF receptors retain specificity for EGF; modified EGF receptors form dimers in the presence of EGF; and MRBLs bearing these receptors exhibit sensitivity to EGF in solution. These results indicated that, in the presence of EGF, MRBL-borne modified EGF receptors form dimers which increase the MRBL permeability to encapsulated compounds (Figure 4).

## Discussion

This work demonstrates that the transmembrane incorporation of EGF receptors covalently crosslinked to p-toluic acid or methyl-PEO_4_-NHS ester in MRBLs makes these MRBLs sensitive to EGF in solution. Specifically, when drug compounds are encapsulated in these MRBLs, a higher rate of *in vitro* drug release from these MRBLs is observed when EGF is present (versus when EGF is not present) in solution. EGF receptors modified with the crosslinking procedure described herein retain the ability to form dimers in the presence of EGF. In addition, when these modified EGF receptors are reconstituted in MRBLs, the MRBLs demonstrate the ability to bind EGF specifically.

The results imply that modified EGF receptors reconstituted in MRBLs bind to EGF (if present) in the solution and (if EGF is present in the solution) form dimers which increase MRBL permeability to encapsulated compounds. Further work may be performed to elucidate the precise mechanism whereby MRBL-reconstituted modified EGF receptor dimers increase the MRBL permeability.

## Methods

### Methods summary

Covalent crosslinking procedures have been described previously [14], [15]. For the preparation and collection of MRBLs, lipid film hydration of egg phosphatidylcholine followed by dialysis and sucrose gradient centrifugation was used. The dimerization capability of modified EGF receptors was assayed by SDS-PAGE electrophoresis as described previously [23]. Reconstituted receptor binding specificity was evaluated using radiolabeled [^125^I]EGF as described [16]. For assessment of drug release from MRBLs, ^1^H NMR spectroscopy (400 MHz, CDCl_3_) was used to evaluate the increase in amount of released drug in a sample over time [24], [25], [26].

### Modification of receptors

Methyl-PEO_4_-NHS ester (an NHS-activated polyethylene oxide compound) (Pierce) was added at a molar ratio of 1∶1 to epidermal growth factor receptor in 50 mM Tris-HCl, 150 mM NaCl, 0.5 mM EDTA, 0.02% Triton X-100, 2 mM DTT, 50% glycerol, pH 7.5 (Invitrogen), and the solution was thoroughly mixed. Separately, p-toluic acid (Alfa Aesar) was added at a molar ratio of 1∶1 and crosslinked to (unmodified) EGF receptor in 50 mM Tris-HCl, 150 mM NaCl, 0.5 mM EDTA, 0.02% Triton X-100, 2 mM DTT, 50% glycerol, pH 7.5 by using EDC and NHS (Pierce) [14], [15]. The two separate solutions were mixed to obtain a single modified EGF receptor solution.

### Preparation of liposomes

Egg phosphatidylcholine (98%; Lipoid) was dissolved in chloroform in a polypropylene tube. Chloroform was evaporated with a stream of nitrogen, leaving a thin lipid film, which was redried under a stream of nitrogen to remove residual traces of solvent [16], [17], [18], [19], [20]. The lipid film was rehydrated in Tris-buffered saline (20 mM Tris, 150 mM NaCl, pH 7.4) containing 50 mM octyl-β-glucoside (Pierce) and thoroughly mixed by vortexing. The solution was dialyzed for 36 h against three changes of buffer (Tris-buffered saline, 30 mM benzamidine (Calbiochem), 0.1 mM PMSF (Pierce)) to remove the detergent, allowing liposomes to form. The resulting turbid solution was mixed with sucrose to 40% (w/v) and applied at the bottom of a sucrose gradient (0.5 mL 40% sucrose, 1.5 mL 20% sucrose, 1.5 mL 5% sucrose), then centrifuged at 40,000 g for 3 h to remove residual traces of detergent. Fractions were collected from the top of the gradient. Liposomes were freshly prepared for each experiment [16], [17].

### Preparation of drug-encapsulating liposomes

Drug-encapsulating liposomes were generated by the addition of actinomycin D (EMD Biosciences) to the rehydrated lipid-containing Tris-buffered saline solution prior to vortexing [17].

### Preparation of MRBLs

Modified epidermal growth factor receptor-bearing liposomes (MRBLs) were generated by the addition of modified EGF receptor in 50 mM Tris-HCl, 150 mM NaCl, 0.5 mM EDTA, 0.02% Triton X-100, 2 mM DTT, 50% glycerol, pH 7.5 to the rehydrated lipid-containing Tris-buffered saline solution prior to vortexing [16], [18].

### Dimerization assays for free modified EGFR

Analysis of modified EGF receptor dimerization capability was performed using SDS-PAGE with Coomassie blue staining, exactly as described [21], [22], [23].

### Binding assays for MRBL-borne modified EGFR

Analysis of modified EGF receptor ligand-binding capability was performed using radiolabeled [^125^I]EGF (PerkinElmer), exactly as described [16].

### Drug release assays

*In vitro* release experiments were carried out at an incubation temperature of 298 K by phase separating free drug (i.e., unencapsulated drug) and drug-encapsulating MRBLs (i.e., encapsulated drug) in a sample at various time points and removing small aliquots from the free drug phase of the sample. The change in concentration of unencapsulated drug in the sample over time was determined using ^1^H NMR spectroscopy (400 MHz, CDCl_3_) [24], [25], [26].
